# The health loss from ischemic stroke and intracerebral hemorrhage: evidence from the North East Melbourne Stroke Incidence Study (NEMESIS)

**DOI:** 10.1186/1477-7525-8-49

**Published:** 2010-05-14

**Authors:** Dominique A Cadilhac, Helen M Dewey, Theo Vos, Rob Carter, Amanda G Thrift

**Affiliations:** 1 National Stroke Research Institute, Heidelberg Heights 3081, Vic, Australia; 2 Department of Medicine, The University of Melbourne 3010, Australia; 3 Deakin Health Economics, Deakin University, Burwood 3125, Australia; 4 Department of Neurology, Austin Health, Heidelberg 3084, Australia; 5 School of Population Health, University of Queensland, Herston 4006, Australia; 6 Baker IDI Heart and Diabetes Institute, Melbourne, Australia; 7 Department Epidemiology & Preventive Medicine, Monash University, Melbourne, Australia

## Abstract

**Background:**

People suffering different types of stroke have differing demographic characteristics and survival. However, current estimates of disease burden are based on the same underlying assumptions irrespective of stroke type. We hypothesized that average Quality Adjusted Life Years (QALYs) lost from stroke would be different for ischemic stroke and intracerebral hemorrhage (ICH).

**Methods:**

We used 1 and 5-year data collected from patients with first-ever stroke participating in the North East Melbourne Stroke Incidence Study (NEMESIS). We calculated case fatality rates, health-adjusted life expectancy, and quality-of-life (QoL) weights specific to each age and gender category. Lifetime 'health loss' for first-ever ischemic stroke and ICH surviving 28-days for the 2004 Australian population cohort was then estimated. Multivariable uncertainty analyses and sensitivity analyses (SA) were used to assess the impact of varying input parameters e.g. case fatality and QoL weights.

**Results:**

Paired QoL data at 1 and 5 years were available for 237 NEMESIS participants. Extrapolating NEMESIS rates, 31,539 first-ever strokes were expected for Australia in 2004. Average discounted (3%) QALYs lost per first-ever stroke were estimated to be 5.09 (SD 0.20; SA 5.49) for ischemic stroke (n = 27,660) and 6.17 (SD 0.26; SA 6.45) for ICH (n = 4,291; p < 0.001). QALYs lost also differed according to gender for both subtypes (ischemic stroke: males 4.69 SD 0.38, females 5.51 SD 0.46; ICH: males 5.82 SD 0.67, females 6.50 SD 0.40).

**Discussion:**

People with ICH incurred greater loss of health over a lifetime than people with ischemic stroke. This is explained by greater stroke related case fatality at a younger age, but longer life expectancy with disability after the first 12 months for people with ICH. Thus, studies of disease burden in stroke should account for these differences between subtype and gender. Otherwise, in countries where ICH is more common, health loss for stroke may be underestimated. Similar to other studies of this type, the generalisability of the results may be limited. Sensitivity and uncertainty analyses were used to provide a plausible range of variation for Australia. In countries with demographic and life expectancy characteristics comparable to Australia, our QoL weights may be reasonably applicable.

## Background

Worldwide, stroke is a significant contributor to disease burden. In Australia, stroke is the second leading cause of death [1]. Of those with first-ever stroke, about 35% die within 12 months of their stroke [2] and about half of survivors at 12 months are dependent on others [3]. The cost of stroke is high, with the present value of lifetime costs of first-ever stroke in 2004 estimated to cost more than AUD2 billion (~2% of total annual health expenditure [4]) to the Australian community [5].

In the NEMESIS study, 72.5% of strokes were ischemic stroke while 14.5% were intracerebral hemorrhage (ICH); 4.3% were subarachnoid hemorrhage and in 8.7% the subtype was undetermined [6]. Importantly, these different types of stroke have different risk factors, treatments and outcome. Case-fatality at one year is lower for first-ever ischemic stroke (reported range from Australian incidence studies 23% to 31%) than for ICH (39% to 50%) and undetermined stroke (67% to 89%) [6,7]. When assessing the effect of stroke on society it is important to include measures of both mortality and morbidity, as stroke affects both of these outcomes.

Summary measures of population health include Health Adjusted Life Expectancy (HALE), Disability Adjusted Life Years (DALYs) and Quality Adjusted Life Years (QALYs). The HALE value represents the number of expected years of life equivalent to years lived in full health adjusted for time spent in poor health based on current rates of ill-health (e.g. chronic disease) and mortality in a community. The DALY is a health gap measure and captures the years of life lost (YLL) due to premature mortality (in this case from stroke) and the years of life lived with disability (YLD) as a consequence of having had a stroke. QALYs are based on a similar conceptual framework (life expectancy plus quality of life [QoL]), but are often based on different assumptions and methods [8,9]. DALYS have gained prominence in recent years with the extensive systematic review of burden of disease [10].

One of the main differences for DALYs and QALYS is the way health states (i.e. the physical, social and emotional functioning of individuals) [11], such as stroke, are weighted. Traditionally, QALYs are based on a heath related QoL weight that is ***directly derived from patients or the general population*** while DALY weights have more commonly been ***elicited from expert panels***[8,12]. There are both advantages and disadvantages for each approach. Regardless, the preference weights derived for both DALYs and QALYs reflect departures from good health [8,13]. It has been found that stroke survivors usually assign themselves higher utilities (i.e. a value that represents the strength of an individual's preference for a particular health outcome) than do the general community or caregivers of stroke survivors [8]. Current estimates of DALYs for stroke in Australia have used preference weights directly elicited from patients [14].

QALYs are usually reported as something society will want to gain, while DALYs are to be avoided. To keep the concept similar to a DALY, one can instead report the QALYs lost. This provides a measure of the health gap experienced by stroke survivors compared to the normal population and provides an estimate of the health gain that could be achieved if a stroke was prevented. Such information is important when making assessments of the value of various interventions, such as in cost-effectiveness analysis.

Health-related QoL of stroke patients has been well documented as part of the North East Melbourne Stroke Incidence Study (NEMESIS). This provided an opportunity to estimate the health loss attributable to stroke using prospectively elicited QoL data from individual stroke patients. In NEMESIS a health state classification questionnaire, the Assessment of Quality of Life (AQoL) tool [15], was used whereby a utility score (an index of the strength of a person's preference for a health state ***where death is represented as 0.0 and normal health as 1.0***) can be derived. This utility score can be used to provide the preference weight in estimating health loss. Because of the recognized differences between the main stroke subtypes, we hypothesized that average QALYs lost would be different for ischemic stroke and ICH.

## Methods

We developed a 'Lifetable' model, created in Excel (Microsoft Corporation, 2003), to accommodate a 'lifetime' perspective for an incident cohort of people with stroke.

Incidence and case-fatality data obtained from NEMESIS were applied to Australian population data to estimate the size of the cohort. The NEMESIS data constitute 'best available' information. Authors of the 2003 Australian BoD study have used NEMESIS data and have compared representative Australian hospital admission rates from 1996/1997 with those in NEMESIS mapped to the relevant statistical local areas[14]. The ratios of hospital admissions in NEMESIS catchment areas to those in all of Australia in 1996/97 ranged from 0.9 to 1.3 by age and sex with an overall ratio close to unity (personal communication, T. Vos March 2010). Nonetheless, we included point estimates used from NEMESIS in the sensitivity and uncertainty analyses as outlined in the Analysis section below. In addition, because early mortality in ICH is much greater than in ischemic stroke and would dominate comparisons with ischemic stroke, estimates of health loss were based on 28 day survivors. Twenty-eight day survivors were categorized into age-groups according to their starting age (mid-point ages for < 55, 55 to 64, 65 to 74, 75 to 84 and 85+ years) up to age 100. Deaths attributable to stroke (not 'all cause' mortality) were calculated for day 28, 12 months and then for each year up to 5 years after stroke using NEMESIS data. Between 5 and 10 years the probability of dying each year attributable to stroke was maintained at the same year 5 probability since no other data were available. After 10 years, remaining survivors were assumed to have the same probability of death as the general population for that age band and gender. Although one may argue that mortality risk may not return to that of the general population, since risk factors in the general population will have increased over time, and may be at similar levels to those of the stroke population, we therefore felt this was a plausible approach given the current limitations of data.

The QoL weights used to estimate QALYs lost were derived from the AQoL Mark 1 instrument which has previously been validated for use in stroke using NEMESIS data [16]. We used published 'normal' population values derived from the AQoL instrument [17] by age band as a measure of pre-stroke QoL. The QoL (*preference*) weight was then calculated as the net difference between these two scores, and could be described as the ***utility loss attributable to stroke***. Because it was important that change in QALYs over time be reported using longitudinal data we used the average of paired utilities obtained from the same cases at 12 months and 5 years. In applying these utility values in the 'Lifetable' model a linear relationship between the 12 month and 5 year values were assumed. This is because the direction and magnitude of change between the 12 month and 5 year time points for each age band varied and was usually small (min 0.006 in < 55 year olds and max 0.12 in 75-84 year olds). Therefore, a more elaborate approach was considered unnecessary. After 5 years, we assumed that survivors would have the same utility loss as the 5 year utility loss in each relevant age band.

To avoid overestimating life expectancy, we used HALE values calculated for the Australian 2003 BoD study [18] for life expectancy in our Lifetable model according to age group. That is, the net difference in health loss due to stroke was estimated by subtracting the estimated HALE value for someone without stroke from that estimated for a person of the same age and gender with stroke to provide the final QALY loss result. We assumed HALE values for the 2003 population were applicable to the 2004 population used in this analysis. Thus, results from this research could be used for economic evaluations using our redeveloped stroke economic model [5]. Standard population life expectancies were not used as they were considered insufficient to address the impact on QoL, since people with stroke may also have non stroke-related disability as they get older.

### Analysis

T-tests for continuous variables were applied using Intercooled STATA version 8 (Stata Corporation, 2003). The level of significance was set at p < 0.05 (two-sided).

We used a 3% discount rate to accord with methods recommended in the Australian BoD study [14]. Since the issue of discounting QALYs is still debated [19], undiscounted QALYs were also estimated. We also provide estimates for different age groups and gender using a 5% discount rate to accord with other studies. Multivariable probabilistic uncertainty analyses were undertaken using @Risk software version 4.5 (Palisade Corporation, 2005). The sampling variations incorporated for point estimates were based on triangular distributions (minimum, most likely and maximum) that approximate a normal distribution. The minimum and maximum values were obtained from literature reviews or best available data and applied as a proportion greater or less than the point estimate. Variables included incidence (range used -5% to +1%) [20,21]; survival (-2% to +1%) [22,23]; and average QALYs lost (ischemic stroke 4%; ICH 2% based on the plausible variation found using NEMESIS data). Three thousand 'Monte Carlo' simulations were undertaken to ensure convergence. Convergence was defined as less than 1.5% variation in primary outcome statistics, such as numbers of strokes. The 3,000 individually simulated point estimates were used to estimate a mean, median and 95% uncertainty interval for the results.

To test the sensitivity of the QALY loss estimates we substituted the NEMESIS QoL weights and case fatality rates with estimates used in the 2003 BoD study for stroke. To distinguish between these estimates, we define these results as DALYs. The results were compared to describe the potential variation that might occur when different input parameters (e.g. QoL weights and case-fatality rates) for Australia are used.

## Results

At 12 months and 5 years, 237 first-ever stroke survivors provided AQoL responses. The sample size was insufficient to estimate utilities beyond age and gender categories. We found that the utilities obtained for males and females were not statistically different (data not shown). Thus, utility data were disaggregated by age band and the net difference between published normal Australian population data [17] were then estimated (Table 1). In those aged < 64 years, differences between the normal population and NEMESIS survivors were small, and were similar at 12 months and 5 years post stroke. This provides evidence that disutility from stroke tends to stabilise after 12 months in people of working or younger ages. In contrast, in those aged over 64 years, the net difference in utilities between the normal population and NEMESIS survivors was large at both 12 months and 5 years. In addition, the health loss was greater at 5 years than at 12 months and may be associated with survivors experiencing non-stroke related disability.

**Table 1:** Average and net difference in utility scores between Australian population and first-ever stroke survivors.

	Normal population†		NEMESIS utility scores*					Net difference (QoL weights)	
			
Age	n	Mean utility	n	12 months	SD	5 years	SD	12 months	5 years
< 55	578	0.85	27	0.8249	0.2231	0.8189	0.1751	0.0251	0.0311
55-64	378	0.80	33	0.7422	0.2762	0.7553	0.2700	0.0578	0.0447
65-74	301	0.79	85	0.6035	0.2963	0.5485	0.3188	0.1865	0.2415
75-84	263	0.75	74	0.4986	0.3123	0.3722	0.3282	0.2514	0.3778
85+	96	0.66	18	0.2341	0.2643	0.1465	0.2241	0.4259	0.5135
Total	1 616		237					0.1886‡	0.2533‡

*Utility scores were obtained using the Assessment of Quality of Life (AQoL) instrument.

†Obtained from AQoL publication [17] and based on equivalent midpoint age used in the model. The normal population for age less than 55 comprises estimates for the 30-39 age group. This is because the midpoint for NEMESIS cases in the < 55 age group was 33 years. Utility estimates in the other age bands result from normal population for ages 50-59, 60-69, 70-79 and 80+, respectively.

‡summary estimate weighted for age and population size

NEMESIS: North East Melbourne Stroke Incidence Study.

### The estimated 'lifetime' health loss attributable to first-ever strokes

Overall, the average QALYs lost per first-ever stroke case weighted for age and gender distribution was estimated to be 5.09 for ischemic stroke and 6.17 for ICH (Table 2). This means that a person who has a stroke loses 5 or six years of healthy life when compared to the normal population. The equivalent undiscounted weighted average QALYs lost were 7.24 for ischemic stroke and 8.88 for ICH. The effect of a 3% discount rate was to reduce average QALYs lost per case by about two years for ischemic stroke and almost three years for ICH. The difference in average QALYs lost per case was significantly greater for ICH than ischemic stroke (p < 0.0001) and was significantly greater for females than males for each stroke subtypes (both p < 0.0001; Table 2).

**Table 2:** Quality Adjusted Life Years lost according to stroke subtype and gender

	Ischemic stroke		Intracerebral Hemorrhage	
	Males N= 14 139	Females N= 13 521	Males N = 2 087	Females N = 2 204
***QALY lost per case***				
Age Group years	QALYs with 3% discounting (5% discounting, 0% discounting)			
< 55	5.5 (3.7, 11.1)	9.8 (4.7, 20.2)	10.7 (7.3, 21.7)	12.5 (8.6, 25.6)
55-64	6.3 (5.0, 9.7)	7.6 (5.8, 12.1)	7.4 (5.9, 11.0)	10.9 (8.6, 16.3)
65-74	6.6 (5.7, 8.5)	7.6 (6.5, 10.2)	6.6 (5.7, 8.4)	7.6 (6.4, 10.1)
75-84	3.7 (3.3, 4.3)	4.9 (4.4, 5.9)	3.9 (3.5, 4.5)	5.1 (4.6, 6.1)
85+	2.3 (2.1, 2.5)	2.5 (2.4, 2.8)	2.2 (2.1, 2.4)	2.4 (2.2, 2.6)
Weighted average (SD)†	4.69 (0.38)	5.51 (0.46)*	5.82 (0.67)	6.50 (0.40)*
Total weighted average (SD)†	5.09 (0.20)		6.17 (0.26)*	
Undiscounted total weighted average	7.24		8.88	

*p value < 0.0001; QALYs: Quality Adjusted Life Years; SD: standard deviation; †3% discount rate used.

Applying these data to the 2004 Australian population, we estimated that there would be median 27,344 (95% UI 26,517, 27,831) first-ever ischemic strokes and median 4,247 (95% UI 4,121, 4,322) ICHs in Australia. The total QALYs lost attributable to these strokes were 139,018 (95% UI 133,311, 144,574) for incident ischemic stroke and 26,177 (95% UI 25,304, 26,867) for incident ICH (Table 3).

**Table 3:** Modelled estimates of stroke subtype and QALYs lost: Results of uncertainty analyses.

	Modeled point estimates†		95% Uncertainty interval†	
			
Stroke subtype	Mean	Median	Lower bound (2.5%)	Higher bound (97.5%)
*Ischemic stroke*				
Number of strokes	27,291	27,344	26,517	27,831
Total number of QALYs lost (incident cases) *	138,962	139,018	133,311	144,574
*Intracerebral Hemorrhage*				
Number of strokes	4,239	4,247	4,121	4,322
Total number of QALYs lost (incident cases)‡	26,145	26,177	25,304	26,867

QALYs: Quality Adjusted Life Years; *3% discount rate used; †3,000 simulations.

### Sensitivity analysis

**Figure 1:**
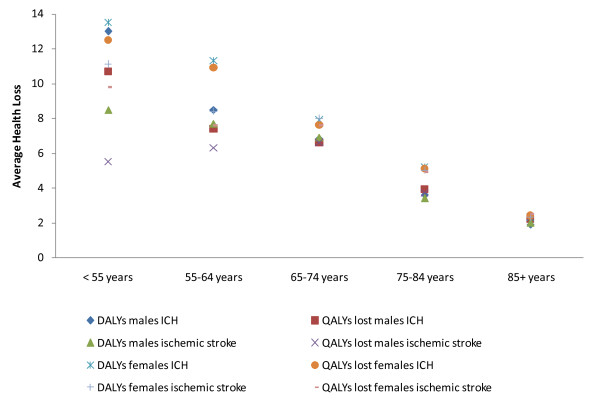
**Differences in average health loss (years) when using different case-fatality and preference disease weights for stroke, according to age group**.

When QoL weights and case fatality rates applied in the 2003 BoD study for stroke were substituted for those used in the primary analysis we found the difference between the two health loss measures (i.e. reported as DALYs and QALYs for simplicity) per case ranged from about 0.28 (~3 months) for ICH and 0.40 (~5 months) for ischemic stroke (Table 4). The greatest differences between the two outcome measures were seen in those aged less than 55-years where the DALY estimate was larger (Figure 1). This difference was observed in both stroke subtypes, among both males (2.3 years for ICH and 3 years for ischemic stroke) and females (1 year for ICH and 1.3 years for ischemic stroke). Conversely, in the older age bands (> 65 years) for males the QALYs lost were slightly greater (approximately 4 months more) than the DALYs.

**Table 4:** Difference in DALYs and QALYs: Sensitivity analyses for average lifetime health loss*.

Stroke subtype	DALYs	QALYs	Difference
Intracerebral hemorrhage (n = 4 291)			
Average per first-ever stroke event	6.45	6.17	0.28
Total health loss for cohort	27 696	26 456	1 240
Ischemic stroke (n = 27 660)			
Average per first-ever stroke event	5.49	5.09	0.40
Total health loss for cohort (years lost)	151 982	140 835	11 147

*Weighted by age and gender distribution of the 2004 reference cohort. DALYs estimated using the Western Australian ' excess' case fatality rate from the WA hospital morbidity database after 28 days following stroke and disability weights for stroke [14]. DALYs: Disability Adjusted Life Years; QALYs: Quality Adjusted Life Years.

## Discussion

We found that people with ICH incurred greater health losses over a lifetime than those having an ischemic stroke and that this is explained by greater stroke-related case fatality in cases with ICH (average over 5 years was 40% greater for ICH). Moreover, since ICH's experience stroke a younger age, those that survive also have a greater duration of disability since these survivors will have a greater life expectancy. We also found that QALY losses were greater for females than males in both stroke subtypes. Previous research on QALYs lost from stroke have been provided for generic stroke or by stroke severity [8,19]. It is reported that only about half of the variance in QoL is explained by stroke severity [13]. Because the QoL weights used were the same for both stroke subtypes and gender, the differences that we observed in QALYs between the stroke types reflect differences in the age of stroke onset and case fatality between these subtypes.

There has been considerable discussion about the best method for deriving the QoL weight. It is important that this is measured carefully because it comprises a major component of the QALY and inaccuracies will lead to inaccurate estimates. The estimates of QoL weights reported in the literature vary considerably for generic stroke, ranging from 0.29 to 0.903 in a recent meta-analysis [13]. These large differences have been attributed to different elicitation methods [19] (e.g. from people with and without stroke or health experts [8]) including the type and range of tool used; timing of elicitation between the event and the assessment; differences in age and gender; and the variance for weights obtained [13]. In our study, the QoL utility loss varied by age and time since stroke and ranged between 0.025 and 0.514. Our summary QoL weight estimate at 12 months was 0.19 and at 5 years 0.25 (adjusted for age and population size). These preference weights are consistent with the lower bound of those reported in the literature.

There are a number of strengths in this study. First, we used comprehensive data obtained from a large community-based stroke incidence study (NEMESIS). Importantly, the QoL weights were directly elicited from the same patients at 12 months and 5 years using the AQoL instrument and are appropriate for the reference population [16]. Use of QoL weights for survivors for up to 5 years is an added advantage to previous studies that have been based on 12-month estimates [13]. This provides a longer-term perspective rarely included in the estimation of health loss for stroke. Furthermore, because health loss was estimated separately for ICH and ischemic stroke, this will enable investigators to evaluate interventions specific to ICH and ischemic stroke.

Similar to other studies of this type, the generalisability of the results may be limited. Although NEMESIS estimates are unbiased at a community level as we have obtained almost every case in a specific population (i.e. community-based rather than hospital-based) and evidence for the 2003 Australian BoD study provide good evidence that these data are fairly representative for Australia as a whole, potential differences across Australia in ethnicity and socioeconomic status may be important. Therefore, it was necessary to provide detailed sensitivity and uncertainty analyses for these data which provide a plausible range of variation for Australia. In countries with demographic and life expectancy characteristics comparable to Australia, our QoL weights should, therefore, be reasonably applicable.

Other limitations include the assumption that NEMESIS incidence rates are applicable for Australia; that use of the normal population utility values to estimate pre-stroke utility are applicable to people who experience stroke when their risk profile may mean they have greater pre-morbid disability; and longer-term (5 year) disutility in people with stroke may reflect co-morbidity from other diseases. Another important limitation was the assumption that in those surviving beyond 5 years the QoL weight values that were applied were those of the next older age band. Because the available longitudinal data re quality of life in the long-term are limited in stroke cohorts we adopted this approach as the most feasible. It also ensured that these data were consistent with those of other studies. Methods used to address this limitation included (1) describing the health loss for stroke according to stroke subtype since this is one of the major factors likely to influence health status over time; (2) use of the 5 year QoL weight values; and (3) multivariable probabilistic uncertainty analysis to assess the impact of a range of important variables including survival rates where evidence is limited but a plausible range of values could be considered. Lastly, the use of triangular distributions rather than use of normal distributions for estimates derived from NEMESIS, such as the QoL weight may have underestimated the uncertainty. It was the considered view of the author group, that triangular distributions would enable ranges to be selected that would best reflect the data we had, as samples size were small when QoL data were disaggregated by the 5 age groups nominated.

These identified limitations may provide sources of over and under estimation of health loss. Inaccurate estimation of health-related QoL can have major implications when used to undertake cost-effectiveness analysis. This is because, as a summary measure for population health which attributes a perceived social value to different health states, variance in QALYs may result in inappropriate resource allocation decisions [19]. In other words, use of different QoL weights may over- or under-estimate the QALY gain for an intervention therefore producing contradictory cost-effectiveness results. Use of multivariable probabilistic uncertainty and sensitivity analyses in this study were used to address such potential limitations and, overall, the estimates were fairly robust.

## Conclusions

Estimates of health loss measured as QALYs were presented for Australia. Notably, these estimates were based on patient-level data obtained up to 5 years following stroke. We provide evidence that the health loss attributable to ICH and ischemic stroke are different; health loss also varies between males and females. Therefore, when undertaking studies of disease burden in stroke, investigators should account for these differences between subtype and gender. Otherwise, in countries where ICH is more common, the disease burden for stroke may be underestimated. Lastly, the estimates provide quantifiable measures of the average health loss over a lifetime per first-ever case of stroke that may be applied in economic evaluations to determine the cost-effectiveness of prevention interventions.

## Competing interests

The authors declare that they have no competing interests.

## Authors' contributions

DC designed the study, contributed to the development of the lifetable models, analysed and interpreted the data and drafted the manuscript. HD participated in the design of the study and helped to draft the manuscript. TV provided access to Australian Burden of Disease data for use in this study, formulated the lifetable model template used in this study and helped to draft the manuscript. RC participated in the design of the study and interpretation of the data and helped to draft the manuscript. AT is the principal investigator for NEMESIS, she contributed to the design of this study and provided input data analysed specifically for this study from NEMESIS related to stroke mortality estimates and paired AQoL utility values at 12 months and 5 years. All authors read and approved the final manuscript.

## Authors' information

All authors have a PhD. DC is the Head of the Public Health Division of the National Stroke Research Institute and has a clinical background in nursing. HD is an Associate Professor for the Department of Medicine (The University of Melbourne) and is the Deputy Director of Neurology at Austin Health (Vic, Australia). TV is the Director, Centre for Burden of Disease and Cost-Effectiveness, School of Population Health at The University of Queensland (Australia) as is also qualified as a medical practitioner. RC is a Professor and the inaugural Chair of Health Economics at Deakin University (Burwood, Australia). AT is an NHMRC Senior Research Fellow, the Head of Stroke Epidemiology at the Baker IDI Heart and Diabetes Institute, as well as an Associate Professor for Monash University (Australia).
